# The Cause of Cancer

**Published:** 1899-07-22

**Authors:** 


### THE CAUSE OF CANCER.

Much has been made during the last week or two over a paragraph which, by some means or another, found access to the general press, in regard to an alleged discovery of the cause of cancer. This was stated to be an injury to the basement membrane, a phrase which it is to be feared the general public found neither comforting nor explanatory. Dr. Lambert Lack has now publicly disclaimed any association with the paragraph in question, and has announced that a short account of his experiments will be published in the "Journal of Pathology," so that we shall soon see what he really means. The idea which has been running in his mind, however, seems to have been to the effect that as cancer cells are practically only normal epithelium out of place, and as from the very commencement they are found growing in lymph spaces, the origin of cancer would be explained by any "injury to the basement membrane" by which the normal epithelium might obtain entrance into the lymph spaces, where it would find no barrier to its continued growth, and would produce all the phenomena of cancer. True; but we know of so many forms of injury to the basement membrane which do not produce cancer that in search for causes we are driven to ask, What is the particular sort of injury which will have this result? and to this we get no answer. Clearly, however, the line of experiment which has been taken up by Dr. Lambert Lack is one of considerable interest. Granting, as we all do, that when once epithelium takes to growing in the lymph spaces we have to do with what is practically a cancer, the question is, Does the cancerous epithelium injure, and thus traverse the basement membrane by virtue of some aggressive power of which it has become possessed—perhaps in consequence of a microbic infection—or does ordinary innocent epithelium when fed by the contents of the lymph spaces develop the vices of the cancer cell? In other words, does the cancer cell differ from the epithelium cell merely by virtue of its environment? Is the intrusion of cancer due to the virulence of the cell, or to the weakness of the protecting basement membrane? The importance of this question is obvious enough; and, although we may fairly doubt whether it is going to be answered by Dr. Lack's simple experiment of placing an emulsion of the epithelial cells of the ovary of a healthy rabbit in the animal's peritoneum, the result which he met with, namely, that on the death of the animal, 14 months later, masses of growth were found in the abdominal and thoracic cavities having the characteristic features of typical ovarian cancer, is a matter of great importance, notwithstanding its premature and irregular announcement.

